# *catena*-Poly[[[diaqua­terbium(III)]-tri-μ_2_-isonicotinato-κ^6^ *O*:*O*′] tris(perchlorate) monohydrate]

**DOI:** 10.1107/S1600536808023623

**Published:** 2008-07-31

**Authors:** Xiao-Hui Huang, Wei-Bo Pan, Xiao-Hong Xu, Rong-Hua Zeng

**Affiliations:** a School of Chemistry and the Environment, South China Normal University, Guangzhou 510006, People’s Republic of China; b South China Normal University, Key Laboratory of the Technology of Electrochemical Energy Storage and Power Generation in Guangdong Universities, Guangzhou 510006, People’s Republic of China

## Abstract

In the title complex, {[Tb(C_6_H_5_NO_2_)_3_(H_2_O)_2_](ClO_4_)_3_·H_2_O}_*n*_, the Tb^III^ ion is coordinated by six O atoms from six isonicotinate (inic) ligands and two water mol­ecules, displaying a bicapped trigonal-prismatic geometry. The inic ligands, which are protonated at the pyridine N atom, link the metal centres, forming a polymeric chain running parallel to the *a* axis. The chains are further assembled *via* intra- and inter­molecular O—H⋯O and N—H⋯O hydrogen-bonding inter­actions into a three-dimensional supra­molecular network involving the inic ligands, the water mol­ecules and the perchlorate anions. One of the perchlorate ions is disordered over two sites with occupancies of 0.561 (17) and 0.439 (17).

## Related literature

For related literature, see: Eddaoudi *et al.* (2001 ▶); Rizk *et al.* (2005 ▶). 
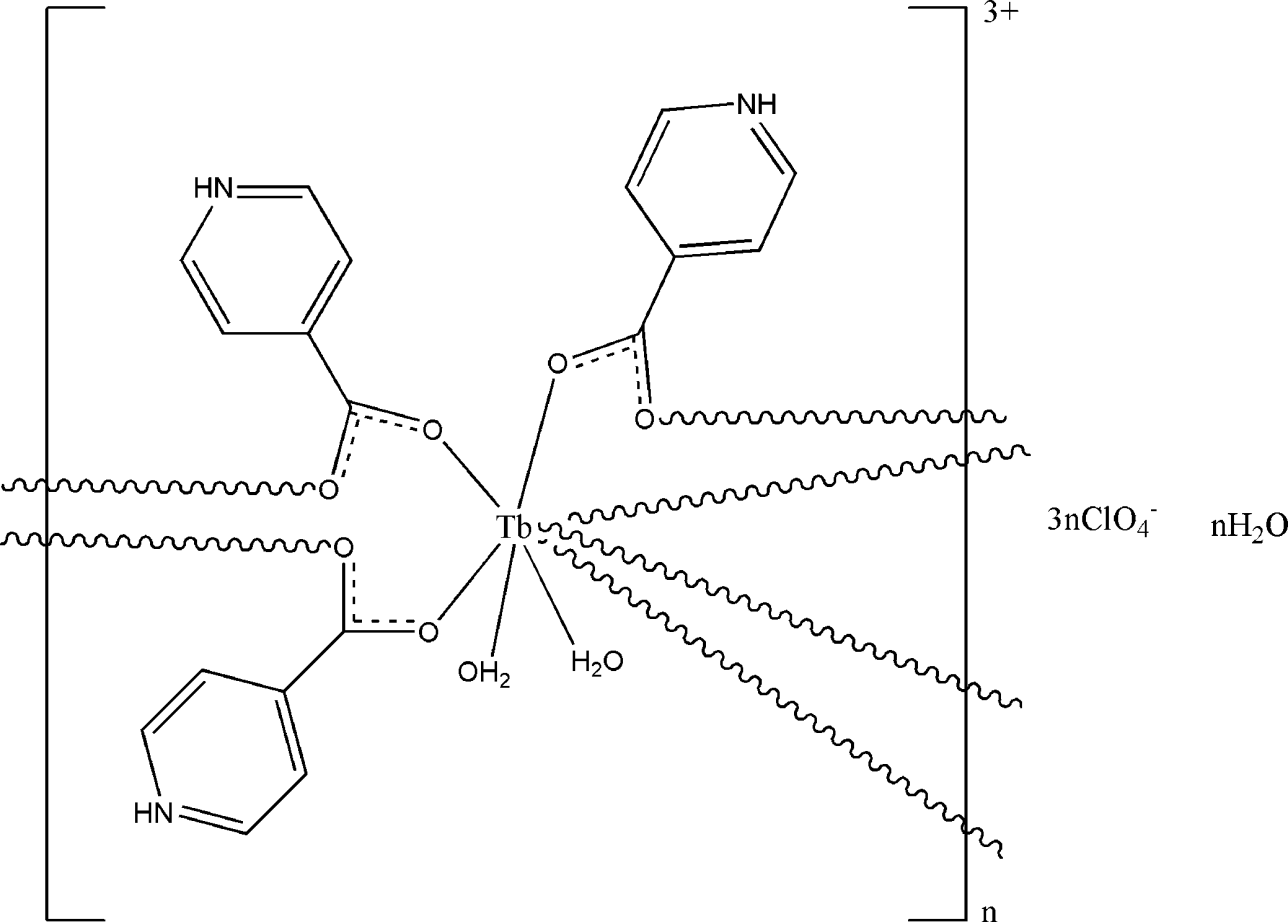


## Experimental

### 

#### Crystal data

[Tb(C_6_H_5_NO_2_)_3_(H_2_O)_2_](ClO_4_)_3_·H_2_O
*M* *_r_* = 880.65
Triclinic, 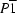

*a* = 9.5270 (4) Å
*b* = 10.9508 (4) Å
*c* = 15.1309 (6) Å
α = 104.402 (2)°
β = 91.480 (2)°
γ = 111.159 (2)°
*V* = 1414.17 (10) Å^3^
*Z* = 2
Mo *K*α radiation
μ = 2.88 mm^−1^
*T* = 296 (2) K
0.20 × 0.18 × 0.15 mm

#### Data collection

Bruker APEXII area-detector diffractometer
Absorption correction: multi-scan (*SADABS*, Sheldrick, 1996 ▶) *T*_min_ = 0.566, *T*_max_ = 0.645
19700 measured reflections
6605 independent reflections
6206 reflections with *I* > 2σ(*I*)
*R*_int_ = 0.024

#### Refinement

*R*[*F* ^2^ > 2σ(*F* ^2^)] = 0.021
*wR*(*F* ^2^) = 0.048
*S* = 1.04
6605 reflections
452 parameters
77 restraints
H-atom parameters constrained
Δρ_max_ = 0.94 e Å^−3^
Δρ_min_ = −0.79 e Å^−3^

### 

Data collection: *APEX2* (Bruker, 2004 ▶); cell refinement: *APEX2*; data reduction: *SAINT* (Bruker, 2004 ▶); program(s) used to solve structure: *SHELXS97* (Sheldrick, 2008 ▶); program(s) used to refine structure: *SHELXL97* (Sheldrick, 2008 ▶); molecular graphics: *SHELXTL* (Sheldrick, 2008 ▶); software used to prepare material for publication: *SHELXL97*.

## Supplementary Material

Crystal structure: contains datablocks I, global. DOI: 10.1107/S1600536808023623/rz2238sup1.cif

Structure factors: contains datablocks I. DOI: 10.1107/S1600536808023623/rz2238Isup2.hkl

Additional supplementary materials:  crystallographic information; 3D view; checkCIF report

## Figures and Tables

**Table 1:** Hydrogen-bond geometry (Å, °)

*D*—H⋯*A*	*D*—H	H⋯*A*	*D*⋯*A*	*D*—H⋯*A*
N1—H1⋯O6^i^	0.86	2.15	2.949 (4)	154
N2—H2⋯O1*W*^ii^	0.86	1.91	2.756 (3)	166
N3—H3*A*⋯O5^iii^	0.86	2.07	2.902 (3)	162
O1*W*—H1*W*⋯O4	0.84	2.48	3.054 (4)	127
O1*W*—H2*W*⋯O13	0.84	2.26	3.030 (3)	152
O2*W*—H4*W*⋯O3*W*^iv^	0.84	2.20	2.920 (3)	145
O2*W*—H4*W*⋯O17	0.84	2.53	3.164 (2)	133
O2*W*—H3*W*⋯O11^v^	0.83	2.23	2.959 (9)	147
O3*W*—H5*W*⋯O12	0.84	2.20	2.934 (9)	146
O3*W*—H6*W*⋯O11^vi^	0.83	2.14	2.843 (9)	142

Symmetry codes: (i) 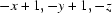; (ii) 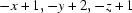; (iii) 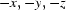; (iv) 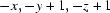; (v) 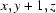; (vi) 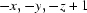.

**Fig. 1.**
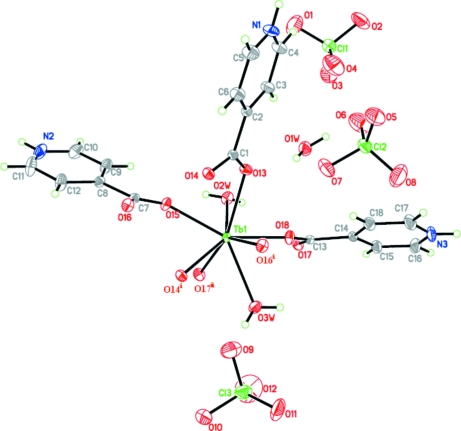
The molecular structure of the title compound showing the atomic-numbering scheme. Displacement ellipsoids drawn at the 30% probability level. Only the major component of disorder is shown. [Symmetry codes: (i) 1-x, 1-y, 1-z; (ii) -x, 1-y, 1-z].

**Fig. 2.**
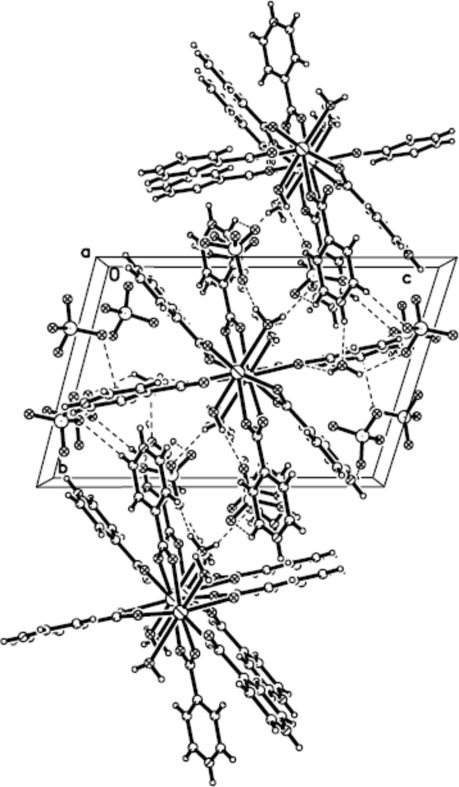
The supramolecular network of the title compound viewed along the a axis. Only the major componentof disorder is shown.

## supplementary crystallographic information

### Comment

The design, synthesis, characterization, and properties of supramolecular networks formed by using functionalized organic molecules as bridges between metal centres are of great interest (Rizk *et al.*, 2005; Eddaoudi *et al.*, 2001). As a building block, isonicotinic acid is an excellent candidate for the construction of supramolecular complexes. Recently, we obtained the title new coordination polymer, whise structure is reported here.

In the title compound, each Tb^III^ centre is coordinated by six oxygen donors of six inic ligands and two water molecules (Fig. 1), and exhibits a bicapped trigonal prismatic coordination geometry. The Tb^III^ ions are linked by inic ligands to form a polymeric chain in the *a* axis direction. The Tb···Tb separations between adjacent metal atoms are 4.318 (4) and 5.259 (5) Å. Intra- and intermolecular O—H···O and N—H···O hydrogen bonding interaction (Table 1) involving the inic ligands, the water molecules and the perchlorate ions assemble neighboring chains into a three-dimensional supramolecular network (Fig. 2).

### Experimental

A mixture of Tb_4_O_7_ (0.189 g, 0.25 mmol), isonicotinic acid (0.135 g, 1.5 mmol) and water (10 ml) in the presence of HClO_4_ (0.385 mmol) was stirred vigorously for 20 min and then sealed into a Teflon-lined stainless-steel autoclave (20 ml capacity). The autoclave was heated to and maintained at 433 K for 3 days, and then cooled to room temperature at 5 K h^-1^ to obtain colourless block-shaped crystals of the title compound suitable for X-ray analysis.

### Refinement

The disordered perchlorate ion was spli into two components with site occupancy factors of 0.561 (17) and0.439 (17). The Cl···O and O···O distances were restrained to be 1.44 (1) and 2.35 (1) Å, respectively. Water H atoms were tentatively located in difference Fourier maps and were refined with distance restraints of O–H = 0.84 Å and H···H = 1.35 Å, and with *U*_iso_(H) = 1.5 *U*_eq_(O). All other H atoms were placed at calculated positions and were treated as riding with C—H = 0.93 Å, N—H = 0.86 Å, and with *U*_iso_(H) = 1.2 *U*_eq_(C, N).

### Crystal data

**Table:**

[Tb(C_6_H_5_NO_2_)_3_(H_2_O)_2_](ClO_4_)_3_·H_2_O	*Z* = 2
*M**_r_* = 880.65	*F*_000_ = 868
Triclinic, *P*1	*D*_x_ = 2.068 Mg m^−^^3^
Hall symbol: -P 1	Mo *K*α radiation λ = 0.71073 Å
*a* = 9.5270 (4) Å	Cell parameters from 6377 reflections
*b* = 10.9508 (4) Å	θ = 1.7–28.0º
*c* = 15.1309 (6) Å	µ = 2.88 mm^−^^1^
α = 104.402 (2)º	*T* = 296 (2) K
β = 91.480 (2)º	Block, colourless
γ = 111.159 (2)º	0.20 × 0.18 × 0.15 mm
*V* = 1414.17 (10) Å^3^	

### Data collection

**Table:**

Bruker APEXII area-detector diffractometer	6605 independent reflections
Radiation source: fine-focus sealed tube	6206 reflections with *I* > 2σ(*I*)
Monochromator: graphite	*R*_int_ = 0.024
*T* = 296(2) K	θ_max_ = 27.8º
φ and ω scan	θ_min_ = 2.1º
Absorption correction: multi-scan(SADABS, Sheldrick, 1996)	*h* = −12→12
*T*_min_ = 0.566, *T*_max_ = 0.646	*k* = −14→14
19700 measured reflections	*l* = −19→19

### Refinement

**Table:**

Refinement on *F*^2^	Secondary atom site location: difference Fourier map
Least-squares matrix: full	Hydrogen site location: inferred from neighbouring sites
*R*[*F*^2^ > 2σ(*F*^2^)] = 0.021	H-atom parameters constrained
*wR*(*F*^2^) = 0.048	*w* = 1/[σ^2^(*F*_o_^2^) + (0.021*P*)^2^ + 1.1255*P*] where *P* = (*F*_o_^2^ + 2*F*_c_^2^)/3
*S* = 1.04	(Δ/σ)_max_ = 0.001
6605 reflections	Δρ_max_ = 0.95 e Å^−^^3^
452 parameters	Δρ_min_ = −0.79 e Å^−^^3^
77 restraints	Extinction correction: none
Primary atom site location: structure-invariant direct methods	

### Special details

**Table:**

Geometry. All e.s.d.'s (except the e.s.d. in the dihedral angle between two l.s. planes) are estimated using the full covariance matrix. The cell e.s.d.'s are taken into account individually in the estimation of e.s.d.'s in distances, angles and torsion angles; correlations between e.s.d.'s in cell parameters are only used when they are defined by crystal symmetry. An approximate (isotropic) treatment of cell e.s.d.'s is used for estimating e.s.d.'s involving l.s. planes.
Refinement. Refinement of *F*^2^ against ALL reflections. The weighted *R*-factor *wR* and goodness of fit *S* are based on *F*^2^, conventional *R*-factors *R* are based on *F*, with *F* set to zero for negative *F*^2^. The threshold expression of *F*^2^ > σ(*F*^2^) is used only for calculating *R*-factors(gt) *etc*. and is not relevant to the choice of reflections for refinement. *R*-factors based on *F*^2^ are statistically about twice as large as those based on *F*, and *R*- factors based on ALL data will be even larger.

### Fractional atomic coordinates and isotropic or equivalent isotropic displacement parameters (Å^2^)

**Table:**

	*x*	*y*	*z*	*U*_iso_*/*U*_eq_	Occ. (<1)
C1	0.5075 (3)	0.5494 (2)	0.34089 (15)	0.0209 (4)	
C2	0.5770 (3)	0.5734 (2)	0.25475 (15)	0.0226 (5)	
C3	0.5067 (3)	0.6111 (3)	0.19077 (17)	0.0316 (5)	
H3	0.4147	0.6209	0.1997	0.038*	
C4	0.5740 (4)	0.6338 (3)	0.11420 (19)	0.0421 (7)	
H4	0.5284	0.6602	0.0712	0.051*	
C5	0.7756 (4)	0.5802 (3)	0.1610 (2)	0.0432 (7)	
H5	0.8660	0.5686	0.1494	0.052*	
C6	0.7134 (3)	0.5586 (3)	0.23913 (19)	0.0331 (6)	
H6	0.7626	0.5341	0.2815	0.040*	
C7	0.5896 (3)	0.7590 (2)	0.58623 (15)	0.0212 (4)	
C8	0.6542 (3)	0.9101 (2)	0.63494 (16)	0.0231 (5)	
C9	0.5830 (3)	0.9941 (3)	0.6193 (2)	0.0358 (6)	
H9	0.4924	0.9581	0.5800	0.043*	
C10	0.6474 (4)	1.1312 (3)	0.6624 (2)	0.0416 (7)	
H10	0.6011	1.1889	0.6520	0.050*	
C11	0.8452 (4)	1.1036 (3)	0.7370 (2)	0.0510 (8)	
H11	0.9340	1.1423	0.7781	0.061*	
C12	0.7861 (3)	0.9652 (3)	0.6950 (2)	0.0386 (7)	
H12	0.8347	0.9101	0.7071	0.046*	
C13	−0.0791 (2)	0.3637 (2)	0.35190 (15)	0.0189 (4)	
C14	−0.1596 (3)	0.2516 (2)	0.26504 (15)	0.0214 (4)	
C15	−0.3153 (3)	0.2093 (3)	0.24151 (18)	0.0316 (6)	
H15	−0.3706	0.2529	0.2772	0.038*	
C16	−0.3860 (4)	0.1024 (3)	0.1649 (2)	0.0432 (7)	
H16	−0.4903	0.0716	0.1489	0.052*	
C17	−0.1552 (4)	0.0837 (3)	0.13243 (19)	0.0426 (7)	
H17	−0.1023	0.0413	0.0934	0.051*	
C18	−0.0790 (3)	0.1880 (3)	0.20941 (17)	0.0314 (5)	
H18	0.0252	0.2155	0.2239	0.038*	
Cl1	0.19389 (9)	0.70180 (8)	0.03011 (5)	0.04494 (17)	
Cl2	0.29246 (9)	0.22081 (7)	0.09772 (5)	0.04040 (16)	
N1	0.7053 (3)	0.6179 (3)	0.10183 (17)	0.0461 (7)	
H1	0.7463	0.6327	0.0536	0.055*	
N2	0.7759 (3)	1.1812 (2)	0.71899 (18)	0.0424 (6)	
H2	0.8156	1.2674	0.7449	0.051*	
N3	−0.3051 (3)	0.0434 (2)	0.11387 (16)	0.0438 (6)	
H3A	−0.3514	−0.0242	0.0665	0.053*	
O1	0.3135 (4)	0.8310 (3)	0.0649 (2)	0.0980 (12)	
O2	0.1590 (4)	0.6721 (3)	−0.06629 (17)	0.0719 (8)	
O3	0.0636 (3)	0.7009 (3)	0.0738 (2)	0.0740 (8)	
O4	0.2370 (4)	0.5967 (3)	0.0487 (2)	0.0825 (9)	
O5	0.4033 (3)	0.2007 (3)	0.03987 (17)	0.0615 (7)	
O6	0.2468 (4)	0.3225 (3)	0.0772 (2)	0.0801 (9)	
O7	0.3532 (4)	0.2641 (3)	0.19129 (16)	0.0728 (8)	
O8	0.1675 (3)	0.0947 (3)	0.0809 (2)	0.0852 (10)	
O13	0.37551 (18)	0.54575 (17)	0.34757 (11)	0.0256 (3)	
O14	0.58826 (19)	0.53420 (17)	0.40081 (11)	0.0272 (4)	
O15	0.45551 (18)	0.70996 (16)	0.54760 (12)	0.0265 (4)	
O16	0.67592 (18)	0.69729 (16)	0.58981 (12)	0.0259 (4)	
O17	−0.14890 (19)	0.43444 (17)	0.39150 (11)	0.0275 (4)	
O18	0.05161 (18)	0.37668 (17)	0.37767 (12)	0.0281 (4)	
Tb1	0.270670 (11)	0.491645 (10)	0.483309 (7)	0.01654 (4)	
O1W	0.1147 (2)	0.53818 (19)	0.22458 (14)	0.0412 (5)	
H1W	0.1025	0.4998	0.1681	0.062*	
H2W	0.1711	0.5100	0.2502	0.062*	
O2W	0.18165 (19)	0.66276 (17)	0.44776 (12)	0.0298 (4)	
H4W	0.0905	0.6479	0.4333	0.045*	
H3W	0.2191	0.7391	0.4861	0.045*	
O3W	0.0974 (2)	0.28637 (18)	0.52869 (13)	0.0323 (4)	
H5W	0.1357	0.2549	0.5636	0.048*	
H6W	0.0490	0.2213	0.4832	0.048*	
Cl3	0.21484 (9)	0.04724 (7)	0.62478 (5)	0.04379 (17)	0.561 (17)
O9	0.3356 (12)	0.1332 (11)	0.5842 (9)	0.062 (3)	0.561 (17)
O10	0.3010 (12)	0.0741 (6)	0.7152 (4)	0.065 (2)	0.561 (17)
O11	0.1764 (12)	−0.0885 (8)	0.5784 (8)	0.066 (2)	0.561 (17)
O12	0.0975 (11)	0.0931 (12)	0.6326 (9)	0.109 (4)	0.561 (17)
Cl3'	0.21484 (9)	0.04724 (7)	0.62478 (5)	0.04379 (17)	0.439 (17)
O9'	0.2951 (16)	0.1488 (12)	0.5860 (10)	0.056 (3)	0.439 (17)
O10'	0.2119 (17)	0.0782 (8)	0.7181 (5)	0.070 (3)	0.439 (17)
O11'	0.2277 (15)	−0.0826 (11)	0.5862 (10)	0.065 (3)	0.439 (17)
O12'	0.0520 (9)	0.0154 (16)	0.5847 (10)	0.108 (4)	0.439 (17)

### Atomic displacement parameters (Å^2^)

**Table:**

	*U*^11^	*U*^22^	*U*^33^	*U*^12^	*U*^13^	*U*^23^
C1	0.0267 (11)	0.0172 (10)	0.0180 (10)	0.0069 (9)	0.0022 (8)	0.0055 (8)
C2	0.0243 (11)	0.0208 (11)	0.0200 (11)	0.0060 (9)	0.0040 (9)	0.0047 (9)
C3	0.0322 (13)	0.0395 (14)	0.0264 (13)	0.0141 (11)	0.0057 (10)	0.0141 (11)
C4	0.0515 (18)	0.0481 (17)	0.0271 (14)	0.0144 (14)	0.0052 (12)	0.0180 (13)
C5	0.0402 (16)	0.0495 (18)	0.0404 (16)	0.0183 (14)	0.0189 (13)	0.0098 (14)
C6	0.0320 (13)	0.0383 (14)	0.0327 (14)	0.0170 (11)	0.0088 (11)	0.0104 (11)
C7	0.0236 (11)	0.0185 (10)	0.0199 (11)	0.0064 (9)	0.0062 (8)	0.0046 (8)
C8	0.0238 (11)	0.0186 (11)	0.0241 (11)	0.0067 (9)	0.0045 (9)	0.0028 (9)
C9	0.0327 (14)	0.0224 (12)	0.0481 (17)	0.0093 (11)	−0.0036 (12)	0.0052 (11)
C10	0.0456 (17)	0.0229 (13)	0.0564 (19)	0.0149 (12)	0.0067 (14)	0.0085 (12)
C11	0.0418 (17)	0.0331 (16)	0.058 (2)	0.0070 (13)	−0.0149 (15)	−0.0099 (14)
C12	0.0378 (15)	0.0268 (13)	0.0439 (16)	0.0129 (11)	−0.0090 (12)	−0.0022 (12)
C13	0.0198 (10)	0.0184 (10)	0.0178 (10)	0.0056 (8)	0.0013 (8)	0.0065 (8)
C14	0.0254 (11)	0.0209 (11)	0.0174 (10)	0.0083 (9)	0.0008 (8)	0.0054 (9)
C15	0.0279 (13)	0.0347 (14)	0.0271 (13)	0.0088 (11)	−0.0016 (10)	0.0050 (11)
C16	0.0391 (16)	0.0383 (16)	0.0384 (16)	0.0031 (13)	−0.0131 (12)	0.0062 (13)
C17	0.065 (2)	0.0353 (15)	0.0265 (14)	0.0237 (14)	0.0060 (13)	−0.0009 (12)
C18	0.0370 (14)	0.0313 (13)	0.0256 (12)	0.0154 (11)	0.0048 (10)	0.0041 (10)
Cl1	0.0505 (4)	0.0462 (4)	0.0342 (4)	0.0140 (3)	0.0078 (3)	0.0105 (3)
Cl2	0.0534 (4)	0.0382 (4)	0.0268 (3)	0.0162 (3)	0.0091 (3)	0.0056 (3)
N1	0.0547 (16)	0.0498 (15)	0.0274 (12)	0.0105 (13)	0.0208 (11)	0.0122 (11)
N2	0.0426 (14)	0.0191 (11)	0.0502 (15)	0.0027 (10)	0.0063 (11)	−0.0033 (10)
N3	0.0626 (17)	0.0297 (12)	0.0244 (12)	0.0088 (12)	−0.0121 (11)	−0.0029 (9)
O1	0.076 (2)	0.072 (2)	0.092 (2)	−0.0128 (16)	0.0228 (18)	−0.0111 (17)
O2	0.122 (2)	0.0698 (18)	0.0360 (13)	0.0448 (17)	0.0139 (14)	0.0228 (12)
O3	0.0541 (15)	0.089 (2)	0.0655 (17)	0.0209 (14)	0.0186 (13)	0.0063 (15)
O4	0.112 (3)	0.099 (2)	0.0642 (18)	0.065 (2)	0.0037 (17)	0.0356 (17)
O5	0.0657 (16)	0.0725 (17)	0.0480 (14)	0.0280 (13)	0.0226 (12)	0.0155 (12)
O6	0.116 (3)	0.091 (2)	0.0670 (18)	0.068 (2)	0.0267 (17)	0.0357 (17)
O7	0.112 (2)	0.0620 (16)	0.0280 (12)	0.0186 (16)	0.0000 (13)	0.0070 (11)
O8	0.0703 (19)	0.0567 (17)	0.089 (2)	−0.0050 (14)	0.0157 (16)	−0.0055 (15)
O13	0.0243 (8)	0.0332 (9)	0.0235 (8)	0.0135 (7)	0.0070 (6)	0.0111 (7)
O14	0.0298 (9)	0.0293 (9)	0.0227 (8)	0.0099 (7)	−0.0030 (7)	0.0102 (7)
O15	0.0225 (8)	0.0180 (8)	0.0336 (9)	0.0046 (6)	−0.0021 (7)	0.0027 (7)
O16	0.0263 (9)	0.0224 (8)	0.0297 (9)	0.0125 (7)	0.0043 (7)	0.0034 (7)
O17	0.0313 (9)	0.0303 (9)	0.0231 (8)	0.0173 (7)	0.0062 (7)	0.0026 (7)
O18	0.0218 (8)	0.0278 (9)	0.0296 (9)	0.0086 (7)	−0.0055 (7)	0.0014 (7)
Tb1	0.01613 (6)	0.01654 (6)	0.01645 (6)	0.00657 (4)	0.00083 (4)	0.00326 (4)
O1W	0.0515 (12)	0.0314 (10)	0.0364 (11)	0.0131 (9)	−0.0026 (9)	0.0068 (8)
O2W	0.0266 (9)	0.0266 (9)	0.0377 (10)	0.0130 (7)	0.0011 (7)	0.0073 (8)
O3W	0.0319 (9)	0.0255 (9)	0.0367 (10)	0.0071 (7)	0.0033 (8)	0.0094 (8)
Cl3	0.0564 (4)	0.0332 (3)	0.0465 (4)	0.0181 (3)	0.0120 (3)	0.0168 (3)
O9	0.067 (5)	0.052 (4)	0.069 (4)	0.017 (3)	0.020 (3)	0.026 (3)
O10	0.092 (5)	0.047 (3)	0.042 (3)	0.007 (3)	−0.009 (3)	0.018 (2)
O11	0.069 (5)	0.027 (3)	0.081 (4)	0.006 (3)	−0.027 (4)	−0.001 (2)
O12	0.083 (5)	0.120 (7)	0.144 (7)	0.066 (5)	0.042 (5)	0.029 (5)
Cl3'	0.0564 (4)	0.0332 (3)	0.0465 (4)	0.0181 (3)	0.0120 (3)	0.0168 (3)
O9'	0.075 (6)	0.035 (4)	0.056 (4)	0.012 (4)	0.018 (4)	0.024 (3)
O10'	0.101 (7)	0.052 (4)	0.049 (4)	0.020 (4)	0.018 (4)	0.016 (3)
O11'	0.067 (6)	0.041 (4)	0.078 (6)	0.022 (4)	−0.002 (5)	0.000 (3)
O12'	0.063 (5)	0.128 (8)	0.129 (8)	0.051 (5)	0.002 (5)	0.008 (6)

### Geometric parameters (Å, °)

**Table:**

C1—O13	1.251 (3)	Cl1—O2	1.416 (3)
C1—O14	1.255 (3)	Cl1—O1	1.419 (3)
C1—C2	1.515 (3)	Cl1—O3	1.419 (3)
C2—C6	1.385 (3)	Cl1—O4	1.438 (3)
C2—C3	1.386 (3)	Cl2—O7	1.411 (2)
C3—C4	1.372 (4)	Cl2—O8	1.419 (3)
C3—H3	0.9300	Cl2—O6	1.428 (3)
C4—N1	1.336 (4)	Cl2—O5	1.432 (2)
C4—H4	0.9300	N1—H1	0.8600
C5—N1	1.332 (4)	N2—H2	0.8600
C5—C6	1.370 (4)	N3—H3A	0.8600
C5—H5	0.9300	O13—Tb1	2.4189 (16)
C6—H6	0.9300	O14—Tb1^i^	2.3152 (16)
C7—O16	1.246 (3)	O15—Tb1	2.3406 (15)
C7—O15	1.254 (3)	O16—Tb1^i^	2.3268 (16)
C7—C8	1.515 (3)	O17—Tb1^ii^	2.3702 (16)
C8—C12	1.375 (4)	O18—Tb1	2.3293 (15)
C8—C9	1.383 (4)	Tb1—O14^i^	2.3152 (16)
C9—C10	1.372 (4)	Tb1—O16^i^	2.3268 (16)
C9—H9	0.9300	Tb1—O17^ii^	2.3701 (16)
C10—N2	1.327 (4)	Tb1—O2W	2.4789 (17)
C10—H10	0.9300	Tb1—O3W	2.5292 (17)
C11—N2	1.321 (4)	O1W—H1W	0.8376
C11—C12	1.380 (4)	O1W—H2W	0.8389
C11—H11	0.9300	O2W—H4W	0.8361
C12—H12	0.9300	O2W—H3W	0.8339
C13—O18	1.242 (3)	O3W—H5W	0.8383
C13—O17	1.250 (3)	O3W—H6W	0.8343
C13—C14	1.512 (3)	Cl3—O10'	1.371 (7)
C14—C18	1.382 (3)	Cl3—O12	1.378 (6)
C14—C15	1.391 (3)	Cl3—O9'	1.378 (9)
C15—C16	1.372 (4)	Cl3—O11	1.382 (8)
C15—H15	0.9300	Cl3—O11'	1.447 (9)
C16—N3	1.323 (4)	Cl3—O9	1.459 (8)
C16—H16	0.9300	Cl3—O10	1.484 (6)
C17—N3	1.333 (4)	Cl3—O12'	1.534 (8)
C17—C18	1.373 (4)	O10—O10'	0.867 (9)
C17—H17	0.9300	O12—O12'	0.921 (10)
C18—H18	0.9300	O12—O10'	1.745 (12)
			
O13—C1—O14	124.7 (2)	C17—N3—H3A	118.5
O13—C1—C2	118.7 (2)	C1—O13—Tb1	115.63 (14)
O14—C1—C2	116.6 (2)	C1—O14—Tb1^i^	177.12 (16)
C6—C2—C3	118.9 (2)	C7—O15—Tb1	136.30 (15)
C6—C2—C1	120.1 (2)	C7—O16—Tb1^i^	144.47 (15)
C3—C2—C1	121.0 (2)	C13—O17—Tb1^ii^	152.03 (16)
C4—C3—C2	119.5 (3)	C13—O18—Tb1	148.90 (15)
C4—C3—H3	120.3	O14^i^—Tb1—O16^i^	76.86 (6)
C2—C3—H3	120.3	O14^i^—Tb1—O18	142.30 (6)
N1—C4—C3	119.6 (3)	O16^i^—Tb1—O18	81.76 (6)
N1—C4—H4	120.2	O14^i^—Tb1—O15	75.81 (6)
C3—C4—H4	120.2	O16^i^—Tb1—O15	124.12 (6)
N1—C5—C6	119.4 (3)	O18—Tb1—O15	141.36 (6)
N1—C5—H5	120.3	O14^i^—Tb1—O17^ii^	81.73 (6)
C6—C5—H5	120.3	O16^i^—Tb1—O17^ii^	140.19 (6)
C5—C6—C2	119.8 (3)	O18—Tb1—O17^ii^	95.97 (6)
C5—C6—H6	120.1	O15—Tb1—O17^ii^	81.27 (6)
C2—C6—H6	120.1	O14^i^—Tb1—O13	122.25 (6)
O16—C7—O15	127.4 (2)	O16^i^—Tb1—O13	76.08 (6)
O16—C7—C8	116.1 (2)	O18—Tb1—O13	81.06 (6)
O15—C7—C8	116.6 (2)	O15—Tb1—O13	78.95 (6)
C12—C8—C9	119.3 (2)	O17^ii^—Tb1—O13	143.18 (6)
C12—C8—C7	119.7 (2)	O14^i^—Tb1—O2W	140.16 (6)
C9—C8—C7	121.0 (2)	O16^i^—Tb1—O2W	140.79 (6)
C10—C9—C8	119.3 (3)	O18—Tb1—O2W	71.74 (6)
C10—C9—H9	120.3	O15—Tb1—O2W	70.71 (6)
C8—C9—H9	120.3	O17^ii^—Tb1—O2W	72.52 (6)
N2—C10—C9	119.7 (3)	O13—Tb1—O2W	71.74 (6)
N2—C10—H10	120.1	O14^i^—Tb1—O3W	73.90 (6)
C9—C10—H10	120.1	O16^i^—Tb1—O3W	71.06 (6)
N2—C11—C12	120.1 (3)	O18—Tb1—O3W	69.91 (6)
N2—C11—H11	120.0	O15—Tb1—O3W	141.20 (6)
C12—C11—H11	120.0	O17^ii^—Tb1—O3W	70.93 (6)
C8—C12—C11	119.0 (3)	O13—Tb1—O3W	138.50 (6)
C8—C12—H12	120.5	O2W—Tb1—O3W	122.65 (6)
C11—C12—H12	120.5	H1W—O1W—H2W	107.2
O18—C13—O17	125.8 (2)	Tb1—O2W—H4W	123.7
O18—C13—C14	116.4 (2)	Tb1—O2W—H3W	113.6
O17—C13—C14	117.84 (19)	H4W—O2W—H3W	107.2
C18—C14—C15	119.4 (2)	Tb1—O3W—H5W	118.0
C18—C14—C13	120.0 (2)	Tb1—O3W—H6W	112.5
C15—C14—C13	120.6 (2)	H5W—O3W—H6W	106.9
C16—C15—C14	119.1 (3)	O10'—Cl3—O12	78.8 (5)
C16—C15—H15	120.5	O10'—Cl3—O9'	119.3 (7)
C14—C15—H15	120.5	O12—Cl3—O9'	91.0 (7)
N3—C16—C15	119.7 (3)	O10'—Cl3—O11	117.0 (7)
N3—C16—H16	120.2	O12—Cl3—O11	116.2 (5)
C15—C16—H16	120.2	O9'—Cl3—O11	121.0 (9)
N3—C17—C18	119.7 (3)	O10'—Cl3—O11'	113.6 (7)
N3—C17—H17	120.1	O12—Cl3—O11'	135.4 (6)
C18—C17—H17	120.1	O9'—Cl3—O11'	114.8 (8)
C17—C18—C14	119.0 (3)	O10'—Cl3—O9	121.1 (7)
C17—C18—H18	120.5	O12—Cl3—O9	110.0 (5)
C14—C18—H18	120.5	O11—Cl3—O9	110.2 (6)
O2—Cl1—O1	110.4 (2)	O11'—Cl3—O9	99.6 (8)
O2—Cl1—O3	109.95 (19)	O12—Cl3—O10	112.4 (4)
O1—Cl1—O3	109.64 (19)	O9'—Cl3—O10	107.3 (8)
O2—Cl1—O4	108.26 (17)	O11—Cl3—O10	108.0 (5)
O1—Cl1—O4	110.4 (2)	O11'—Cl3—O10	94.6 (6)
O3—Cl1—O4	108.2 (2)	O9—Cl3—O10	98.6 (6)
O7—Cl2—O8	108.89 (19)	O10'—Cl3—O12'	104.2 (6)
O7—Cl2—O6	109.02 (18)	O9'—Cl3—O12'	100.7 (7)
O8—Cl2—O6	111.1 (2)	O11—Cl3—O12'	81.5 (6)
O7—Cl2—O5	110.65 (18)	O11'—Cl3—O12'	100.6 (5)
O8—Cl2—O5	108.17 (17)	O9—Cl3—O12'	116.1 (6)
O6—Cl2—O5	109.06 (17)	O10—Cl3—O12'	138.6 (5)
C5—N1—C4	122.8 (2)	O10'—O10—Cl3	65.4 (6)
C5—N1—H1	118.6	O12'—O12—Cl3	81.1 (7)
C4—N1—H1	118.6	O12'—O12—O10'	115.2 (10)
C11—N2—C10	122.6 (2)	Cl3—O12—O10'	50.4 (3)
C11—N2—H2	118.7	O10—O10'—Cl3	79.5 (7)
C10—N2—H2	118.7	O10—O10'—O12	127.9 (9)
C16—N3—C17	123.1 (2)	Cl3—O10'—O12	50.8 (4)
C16—N3—H3A	118.5	O12—O12'—Cl3	62.5 (6)

Symmetry codes: (i) −*x*+1, −*y*+1, −*z*+1; (ii) −*x*, −*y*+1, −*z*+1.

### Hydrogen-bond geometry (Å, °)

**Table:**

*D*—H···*A*	*D*—H	H···*A*	*D*···*A*	*D*—H···*A*
N1—H1···O6^iii^	0.86	2.15	2.949 (4)	154
N2—H2···O1W^iv^	0.86	1.91	2.756 (3)	166
N3—H3A···O5^v^	0.86	2.07	2.902 (3)	162
O1W—H1W···O4	0.84	2.48	3.054 (4)	127
O1W—H2W···O13	0.84	2.26	3.030 (3)	152
O2W—H4W···O3W^ii^	0.84	2.20	2.920 (3)	145
O2W—H4W···O17	0.84	2.53	3.164 (2)	133
O2W—H3W···O11^vi^	0.83	2.23	2.959 (9)	147
O3W—H5W···O12	0.84	2.20	2.934 (9)	146
O3W—H6W···O11^vii^	0.83	2.14	2.843 (9)	142

Symmetry codes: (iii) −*x*+1, −*y*+1, −*z*; (iv) −*x*+1, −*y*+2, −*z*+1; (v) −*x*, −*y*, −*z*; (ii) −*x*, −*y*+1, −*z*+1; (vi) *x*, *y*+1, *z*; (vii) −*x*, −*y*, −*z*+1.
